# Synthesis of SiO_2_-Al_2_O_3_ Aerogel Powder via Low-Temperature Alkaline Fusion Activation of Potassium Feldspar

**DOI:** 10.3390/gels12080680

**Published:** 2026-08-01

**Authors:** Haoran Qian, Wenjie Cheng, Guiquan Zhou, Junliang Zhang, Song He

**Affiliations:** 1 Mining Engineering Department, Shanxi Institute of Technology, Yangquan 045000, China; 2 School of Safety Science and Emergency Management, Wuhan University of Technology, Luoshi Road 122, Wuhan 430070, China; 13271605410@163.com

**Keywords:** potassium feldspar, low-temperature alkali fusion activation, SiO_2_-Al_2_O_3_ aerogel, thermal stability

## Abstract

This study proposes a process combining alkali-activated potassium feldspar, acid leaching, and sol–gel coupling with supercritical drying to prepare high-performance silica–alumina composite aerogel. The optimal parameters for potassium feldspar alkali-melt activation are a calcination temperature of 350 °C, mass ratio of potassium feldspar to sodium hydroxide of 1:1.2, and calcination time of 120 min, achieving an acid-leaching efficiency of 97.3% for the activated potassium feldspar. The acid leachate, using propylene oxide as a gelling promoter, was processed through aging, solvent exchange, and supercritical drying to yield SiO_2_-Al_2_O_3_ aerogel with typical three-dimensional nanoporous network structure. EDS spectroscopy revealed that the spatial distributions of aluminum and silicon elements were highly coincident and uniformly dispersed. XPS and FTIR further confirmed the formation of Si-O-Al bonds, indicating that aluminum atoms were successfully incorporated into the silico-aluminate tetrahedral network, constructing silicon–aluminum composite framework. The SiO_2_-Al_2_O_3_ aerogel exhibits specific surface area of 660.841 m^2^/g and a pore volume of 1.321 cm^3^/g. Its mass loss within the 0–1000 °C range is only 9.55%, far lower than the 28% mass loss of pure aluminum oxide aerogel, indicating that the silicon–aluminum composite structure effectively suppresses high-temperature phase transitions and framework collapse.

## 1. Introduction

Aerogel is sol–gel material that is dried under strictly controlled conditions to prevent the collapse of its pore structure, resulting in a material containing 90–99% air while retaining an intact solid nanostructure [1,2,3]. First synthesized by Kistle in 1931 using supercritical drying technology [4], aerogels have evolved over the past 90 years into various types, including oxide aerogel, organic aerogel, and carbide aerogel. Aerogels are valued for their low density, high specific surface area, high porosity, low thermal conductivity, and high pore volume [5,6,7,8], aerogels are widely used in numerous fields, including catalysis [9], adsorption [10,11], construction [12,13,14,15], and aerospace [16,17,18].

The preparation process of aerogels primarily includes sol–gel formation, aging, modification, and drying [19,20]. Specifically, colloidal particles in the sol system aggregate to form a wet gel with a three-dimensional, random network structure. The gel is then dried under atmospheric pressure or via supercritical drying, replacing the solvent in the gel network with air while maintaining the integrity of the gel framework [21], thereby producing the aerogel. Whether preparing SiO_2_ aerogels or SiO_2_-Al_2_O_3_ aerogels, the silicon source typically used is ethyl orthosilicate (TEOS) or butyl orthosilicate (TMOS). These silicon sources react with solvents (such as ethanol) or water to form silica gels under appropriate condition [22,23,24,25]. Zhang et al. [26] used MTES and TEOS as co-precursors to rapidly synthesize a dual-pore silica aerogel within 20 h via ambient-pressure drying. Mazraeh et al. [27] prepared aerogels using tetraethyl orthosilicate (TEOS) as the raw material via a two-step catalytic process involving atmospheric-pressure drying under various synthesis conditions. They analyzed the relationship between key process parameters, such as the EtOH/TEOS and H_2_O/EtOH molar ratios, and the synthesis temperature and the micro- and macrostructures of the resulting aerogels. Driven by cost constraints and the concept of sustainable development, research on silica aerogel raw materials has gradually shifted toward the development and utilization of low-cost inorganic silicon sources. Due to its low cost and wide availability, water glass, combined with ion-exchange purification technology and atmospheric pressure drying, has resulted in a significant reduction in the cost of aerogel production. Li et al. [28] used water glass as the raw material, after preparing the gel via the sol–gel method, they performed hydrophobic modification using trimethylchlorosilane (TMCS), and finally obtained hydrophobic SiO_2_ aerogel through atmospheric pressure drying. Currently, the main methods for drying aerogels include supercritical drying, freeze-drying, and atmospheric pressure drying. Supercritical drying uses dry ice or anhydrous ethanol as the supercritical medium to reduce the interfacial tension generated during the phase transition from liquid to gas [29]. The aerogels prepared by this method are typically block-shaped, effectively reducing the damage caused by liquid surface tension to the gel system and maintaining the integrity of the internal structure. Freeze-drying removes liquid from the interior of the gel through sublimation to obtain a dry aerogel [30]. Atmospheric drying is a process in which wet gels are directly dried under atmospheric pressure to obtain aerogel structures [31]. Surface modification is typically employed to control drying shrinkage and minimize damage to the internal structure of the material. Compared to supercritical drying and freeze-drying, atmospheric drying involves relatively simple equipment and operational requirements, lower equipment costs, and faster drying rates, resulting in higher production efficiency. It is currently the most widely used method for preparing aerogels.

In recent years, the preparation of silica aerogels using low-cost materials has attracted widespread attention from researchers. Materials such as kaolin, fly ash, coal gangue, and rice husk ash, which are rich in silicon and aluminum, have been proven to serve as suitable raw materials for aerogel synthesis. The development and utilization of these mineral resources not only effectively reduce raw material costs but also align with the requirements of a circular economy, providing a new approach to the green production of aerogels. Wei et al. [32] prepared a sodium silicate solution using coal gangue as the raw material, then employed a one-pot method to synthesize a wet silica gel. Following solvent exchange, surface modification, and atmospheric drying, they obtained a nano-silica aerogel with an open, porous microstructure. Hu et al. [33] used kaolin as a silicon source. After calcination and activation with sodium carbonate, the material underwent hydrochloric acid dissolution, sodium removal via cation exchange resin, sol–gel processing, and surface modification. and finally dried at atmospheric pressure to produce hydrophobic silica aerogels with low density, high specific surface area, and excellent pore structure, providing a new approach for utilizing abundant and inexpensive kaolin resources to prepare high-performance aerogels. Liu et al. [34] used coal gangue as a raw material. After low-temperature calcination, acid leaching for impurity removal, and alkali fusion activation, they successfully prepared SiO_2_-Al_2_O_3_ composite aerogels using the sol–gel method and vacuum drying. Shen et al. [35] synthesized SiO_2_-Al_2_O_3_ composite aerogels from fly ash using an alkali fusion–acid leaching–atmospheric drying process. Their study found that when the sol’s pH was controlled between 2.7 and 3.1, the resulting aerogels exhibited high specific surface area and good thermal stability. Li et al. [36] used circulating fluidized bed fly ash as a source of silicon and aluminum. Through low-temperature sintering with sodium carbonate, extracting silicon and aluminum via sulfuric acid leaching, followed by surface modification and atmospheric drying, a silicon–aluminum composite aerogel with high specific surface area, excellent hydrophobicity, and superior thermal stability was prepared. When combined with gypsum, it demonstrated excellent thermal insulation properties and mechanical strength, providing a new approach for the high-value utilization of fly ash. Li et al. [37] systematically reviewed valorization strategies for producing high-value advanced ceramics from multi-source solid wastes and natural aluminosilicate resources, identifying low-energy activation and full-component utilization as core goals for sustainable material fabrication. Accordingly, this work employs potassium feldspar as feedstock, develops a 350 °C low-temperature alkali fusion process for efficient silicon and aluminum component extraction, and fabricates SiO_2_-Al_2_O_3_ composite aerogels via sol–gel method combined with supercritical drying. This study provides a new pathway for the high-value utilization of potassium feldspar and enriches the green preparation system of silicon–aluminum composite aerogels.

Current feldspar-based aerogel research is still in its early stage. Existing methods mostly rely on energy-intensive high-temperature calcination to break the crystal lattice, and fail to achieve synergistic Si-Al utilization: aluminum components are lost during leaching, resulting in only pure silica aerogels; some routes require lengthy multi-step alternating acid-base extraction.

Our low-temperature alkali-fusion strategy disrupts the feldspar aluminosilicate framework under mild conditions, enabling efficient simultaneous leaching of silicon and aluminum. The leachate acts as a Si-Al co-precursor and is converted into a 3D Si-O-Al bonded composite network via the sol–gel process, realizing integrated utilization of silicon and aluminum components. Compared with reported feldspar-derived pure silica aerogels, our Si-Al composite aerogel has slightly lower porosity, but its Si-O-Al network significantly inhibits high-temperature framework collapse and phase transition, delivering superior thermal stability. It also achieves full-component high-value utilization of Si and Al in feldspar, breaking the bottleneck of single silicon extraction in existing studies. Due to the characteristics of low activation energy consumption and adjustable purity of potassium feldspar raw material. In this study, potassium feldspar was used as the raw material. Taking advantage of sodium hydroxide’s ability to induce a fusion reaction at relatively low temperatures, the potassium feldspar was subjected to an activation treatment. This process decomposed the originally insoluble potassium-sodium feldspar mineral structure and converted it into the potassium-sodium alunite phase, which is easily soluble under acidic conditions. Building on this, selective extraction of silicon and aluminum was further achieved through an acid leaching process. During the gel preparation stage, propylene oxide was used as a gelation promoter. By leveraging its ring-opening reaction mechanism to regulate the polymerization process of the sol system, the sol system was precisely brought to the critical gelation state. This is followed by key steps such as aging treatment to strengthen the gel network framework and solvent exchange to remove residual impurities and regulate the pore environment. Finally, supercritical drying technology is employed to remove the solvent, resulting in silica–alumina composite aerogel material with three-dimensional nanoporous network structure. This study provides an innovative technical pathway and theoretical basis for the high-value and efficient utilization of potassium feldspar resources, and holds significant scientific and practical value for advancing the deep processing of non-metallic minerals and the green preparation of novel inorganic nanomaterials [38,39].

## 2. Results and Discussion

### 2.1. Analysis of Impurity Removal and Activation of Potassium Feldspar

**Figure 1:**
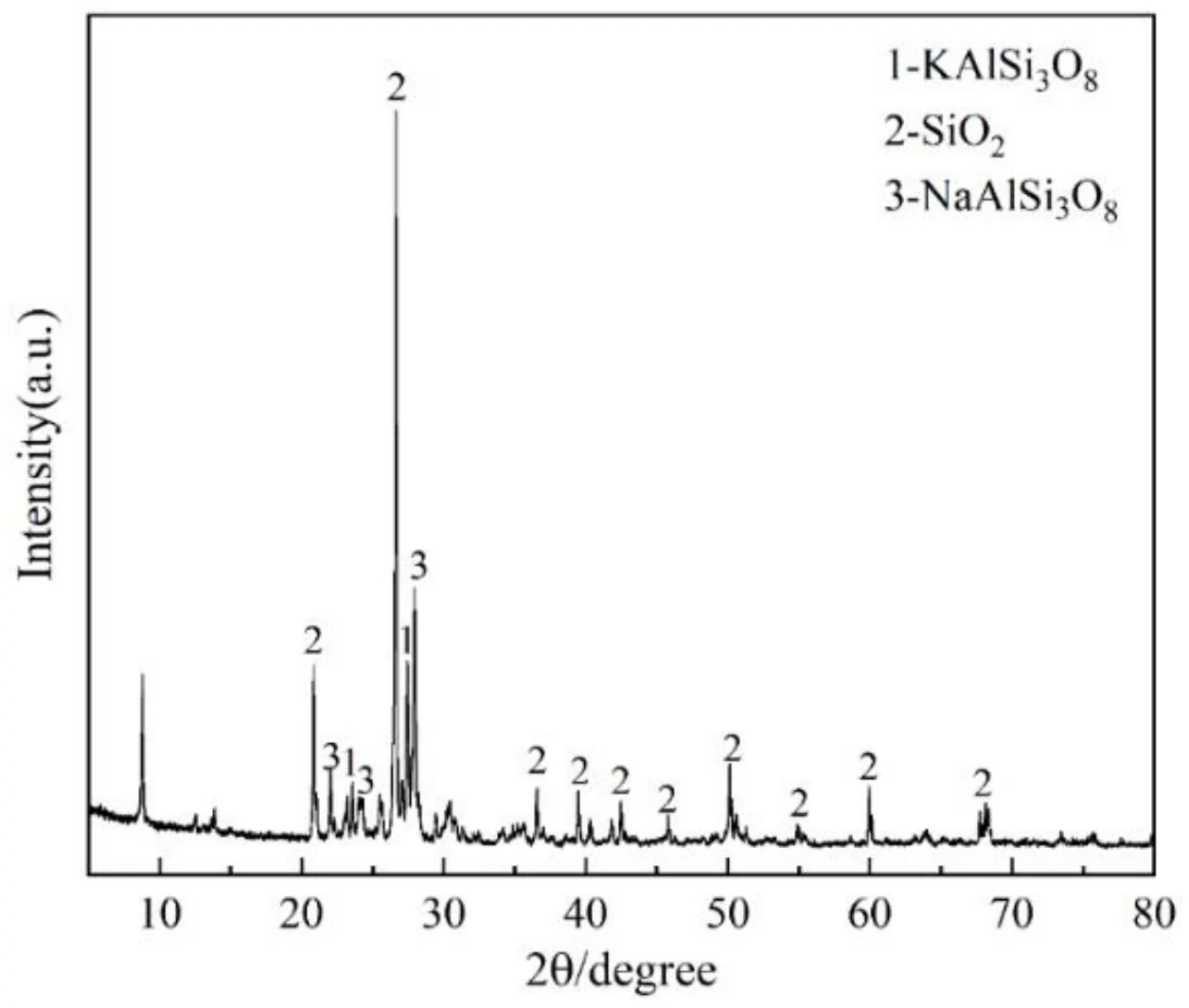
XRD pattern of native potassium feldspar.

Potassium feldspar was used as the raw material for preparing SiO_2_-Al_2_O_3_ aerogel. Table 1 shows the chemical composition of potassium feldspar, which consists primarily of silica and alumina, along with small amounts of metal oxides, SiO_2_ and Al_2_O_3_ account for more than 85% of the total composition. The XRD pattern of potassium feldspar is shown in Figure 1. Based on the diffraction pattern, it can be seen that potassium feldspar contains different mineral phases such as silico-aluminates and quartz. The characteristic diffraction peaks of these mineral phases are clearly visible, indicating that it is feasible to extract silicon and aluminum from potassium feldspar and use them to prepare aerogels.

As shown in Table 1, potassium feldspar contains a certain amount of metal oxide impurities, such as TiO_2_ and Fe_2_O_3_. During subsequent preparation and processing, these metal oxides may exist in solution or within the aerogel samples in ionic form, thereby affecting the aerogel’s performance. Therefore, potassium feldspar can be pretreated to remove these trace amounts of metal oxides [40]. Impurities were removed using a 40% sulfuric acid solution. Potassium feldspar was added to the sulfuric acid solution at a ratio of 1 g per 10 mL. After continuous stirring for 4 h in 90 °C water bath, the mixture was filtered, and the filtrate was washed with water until neutral. The dried filtrate was then used for subsequent calcination and activation.

**Figure 2:**
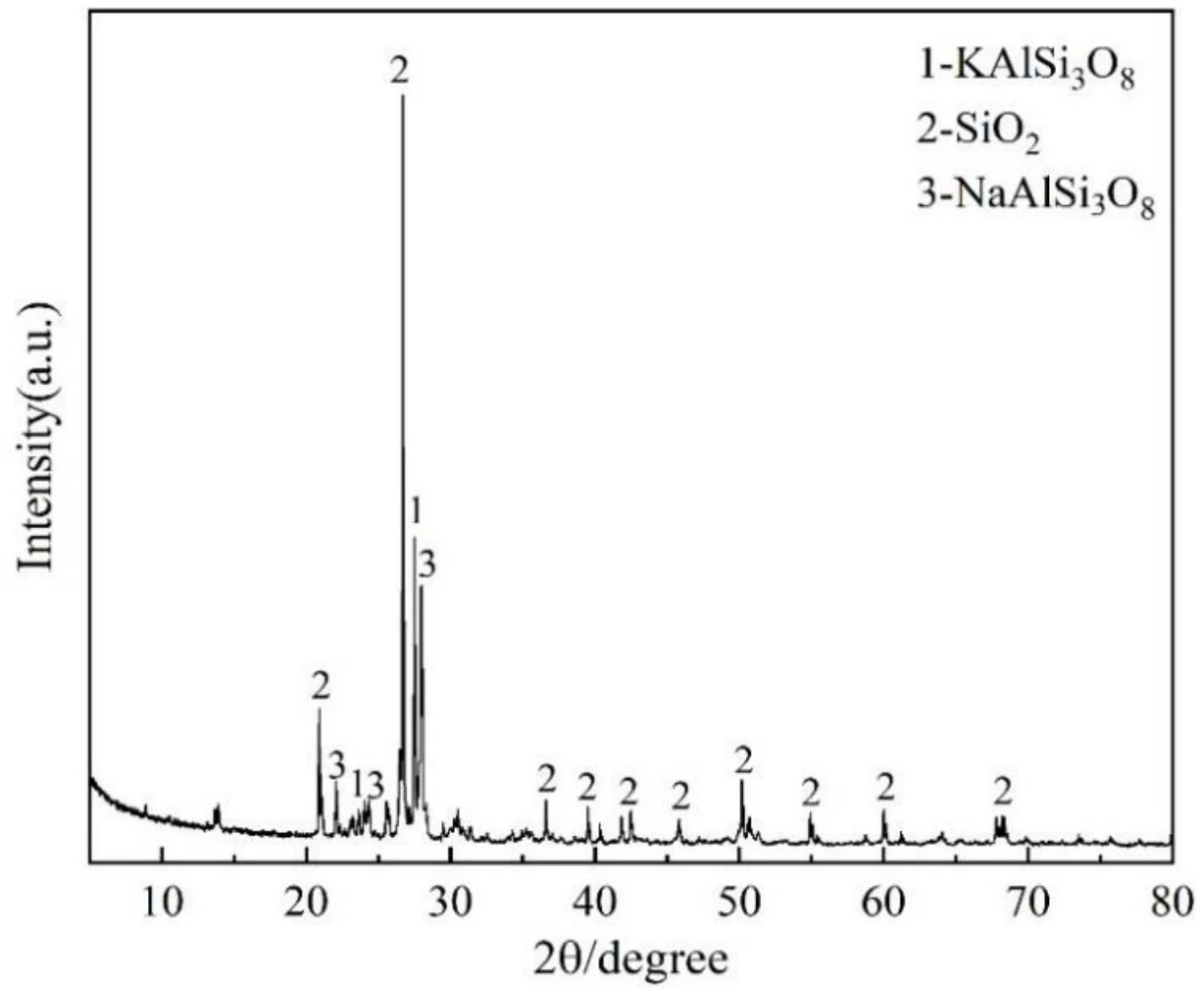
XRD pattern of potassium feldspar after treatment with sulfuric acid.

Table 2 lists the major components of potassium feldspar after sulfuric acid treatment. The Fe_2_O_3_ content decreased from 1.2% to 0.15%, indicating that sulfuric acid pretreatment can remove iron from potassium feldspar. Figure 2 shows the XRD pattern of potassium feldspar after sulfuric acid treatment. The peaks corresponding to the silico-aluminate and quartz phases in the potassium feldspar did not significantly weaken or disappear after treatment, indicating that sulfuric acid does not react with potassium feldspar during the treatment process. Compared to the XRD pattern of the raw potassium feldspar, the diffraction peak at approximately 8.7° in the sulfuric acid-treated sample is significantly weakened. Calculated by Bragg’s law, this peak corresponds to the (001) crystal plane of biotite, a Fe- and Mg-bearing layered aluminosilicate impurity associated with potassium feldspar ore, rather than simple metal oxides such as Fe_2_O_3_ or MgO. The attenuation of this peak indicates that sulfuric acid treatment destroys the layered structure of biotite and leaches out Fe and Mg impurities, achieving the removal of trace metal impurity elements in potassium feldspar [41].

Notably, the removal efficiency of TiO_2_ via sulfuric acid pretreatment is relatively limited, with the content only decreasing from 0.17 wt% to 0.12 wt%. This is because TiO_2_ in potassium feldspar mainly exists as chemically inert rutile microcrystals encapsulated inside mineral particles, which cannot be effectively dissolved by dilute sulfuric acid at 90 °C. The residual trace TiO_2_ (≈0.12 wt%) will be introduced into the final aerogel during the subsequent acid leaching and sol–gel process. Generally, trace TiO_2_ can form a small number of Si-O-Ti bonds in the silicon-oxygen network and slightly improve the framework crosslinking degree, but its effect on the overall pore structure and thermal stability of aerogels is very limited due to the extremely low content.

**Figure 3:**
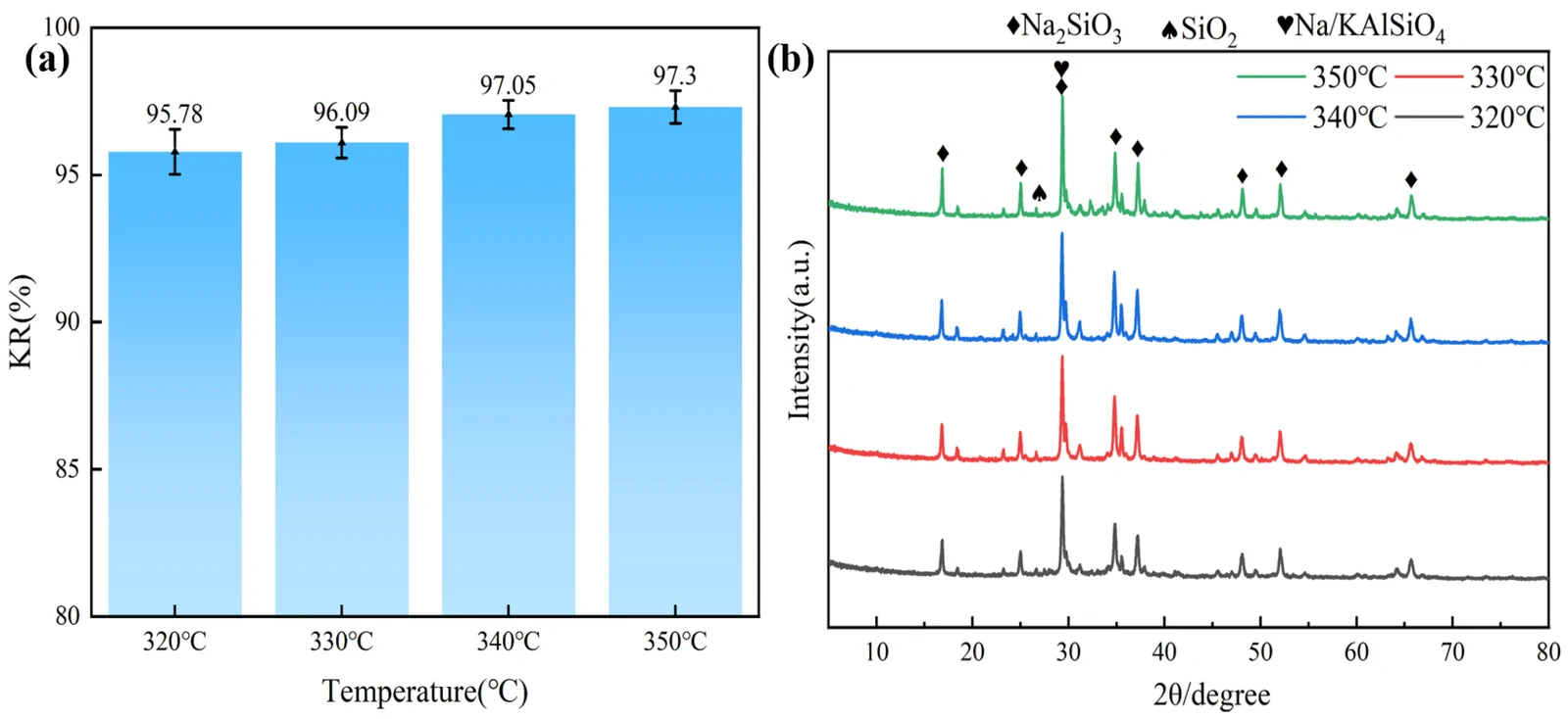
(**a**) Reaction rate (KR) and (**b**) XRD patterns of potassium feldspar at different calcination temperatures.

To fully utilize the silicon and aluminum in potassium feldspar and achieve a high reaction rate KR, three experimental parameters affecting the reaction rate were investigated: calcination temperature, the mass ratio of potassium feldspar to sodium hydroxide (m(potassium feldspar)/m(NaOH)), and calcination time. First, under conditions where m(potassium feldspar)/m(NaOH) was maintained at 1:1.2 and the calcination time was 120 min, the effect of calcination temperature on the reaction rate KR was investigated. The silico-aluminates and quartz in potassium feldspar react with NaOH to form Na_2_SiO_3_ and Na/KAlSiO_4_, which can be dissolved or reacted during subsequent water leaching and acid leaching processes. Figure 3a shows that the reaction rate of potassium feldspar increases with rising temperature. When the temperature rises from 340 °C to 350 °C, the reaction rate increases by only 0.2%, a smaller change compared to the 1.01% increase observed between 330 °C and 340 °C. This indicates that further increases in temperature have a negligible effect on the reaction rate. Moreover, higher temperatures would lead to increased energy consumption. Figure 3b presents the XRD patterns of the activated products at different calcination temperatures. As the temperature increases, the peak intensity of certain components in the products decreases. When the temperature reaches 350 °C, the SiO_2_ peak almost disappears, indicating that the SiO_2_ in the potassium feldspar has been fully converted and utilized to react with sodium hydroxide to form sodium silicate. Therefore, 350 °C is the optimal calcination temperature for the activation of potassium feldspar.

**Figure 4:**
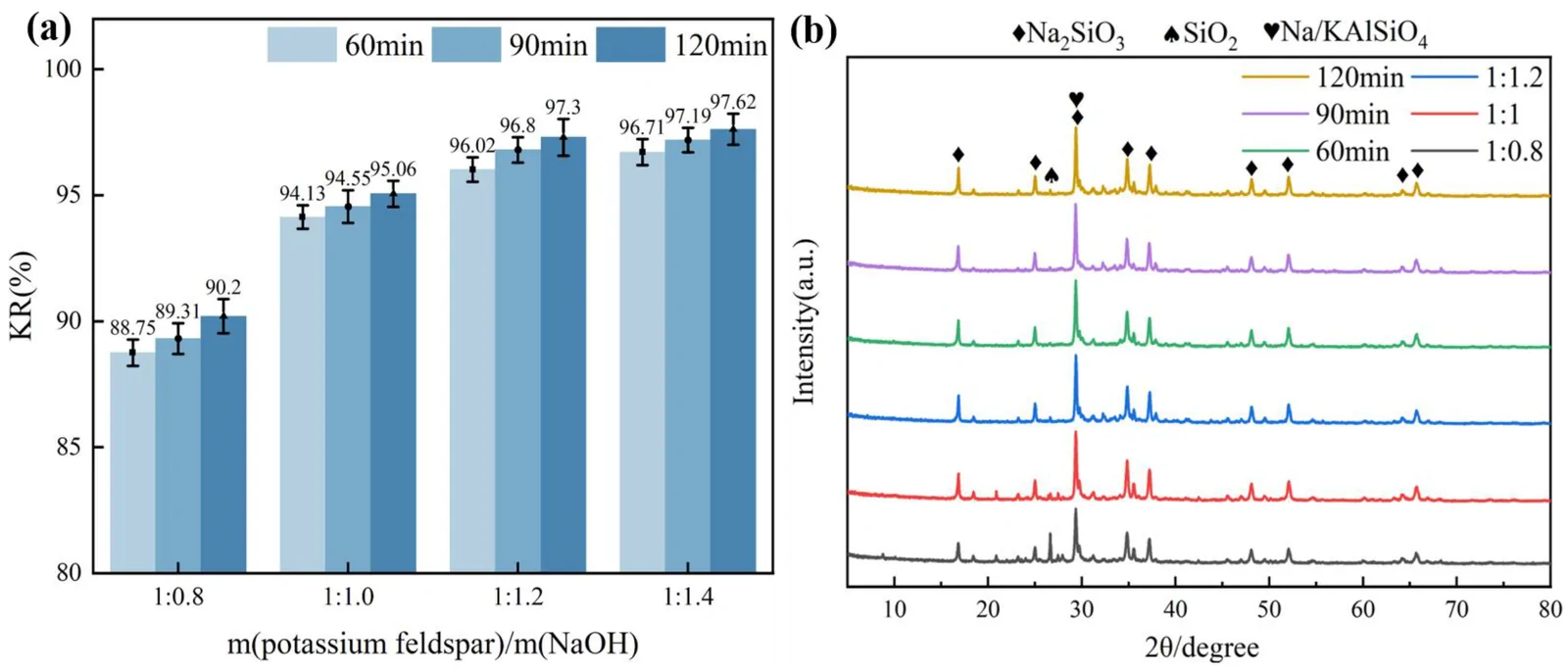
(**a**) Reaction rate (KR) and (**b**) XRD patterns of potassium feldspar at different mass ratios and calcination times.

When the calcination temperature was set to 350 °C, the effect of the mass ratio of potassium feldspar to NaOH on the reaction rate, KR, was investigated. As shown in Figure 4a, the reaction rate of potassium feldspar increased as the mass ratio decreased. When the mass ratio of m(potassium feldspar)/m(NaOH) was 1:1, the reaction rate was 95.06%. When the mass ratio was 1:1.2, the reaction rate reached 97.3%, and when the mass of sodium hydroxide was further increased to a mass ratio of 1:1.4, the reaction rate was 97.62%, representing an increase of only 0.32% compared to the previous value. At the same time, the XRD diffraction pattern shows that the SiO_2_ peak gradually weakens as the mass ratio decreases. A strong SiO_2_ peak is present when the mass ratio of potassium feldspar to sodium hydroxide is 1:0.8, but it virtually disappears at a mass ratio of 1:1.2. Therefore, to ensure that the potassium feldspar is fully reacted and activated while reducing NaOH consumption, the optimal mass ratio of m(potassium feldspar) to m(NaOH) is 1:1.2.

Under conditions of a calcination temperature of 350 °C and a molar ratio of m(potassium feldspar) to m(NaOH) of 1:1.2, the effect of different calcination times on the reaction rate was investigated. As shown in Figure 4, different calcination times had no significant effect on the SiO_2_ peak, and the SiO_2_ in the potassium feldspar was completely reacted. Furthermore, the reaction rate increases with longer calcination times, but the rate of increase gradually decreases. Therefore, 120 min was selected as the optimal calcination time. In summary, the optimal activation conditions for potassium feldspar are a calcination temperature of 350 °C, a molar ratio of potassium feldspar to NaOH of 1:1.2, and a calcination time of 120 min.

**Figure 5:**
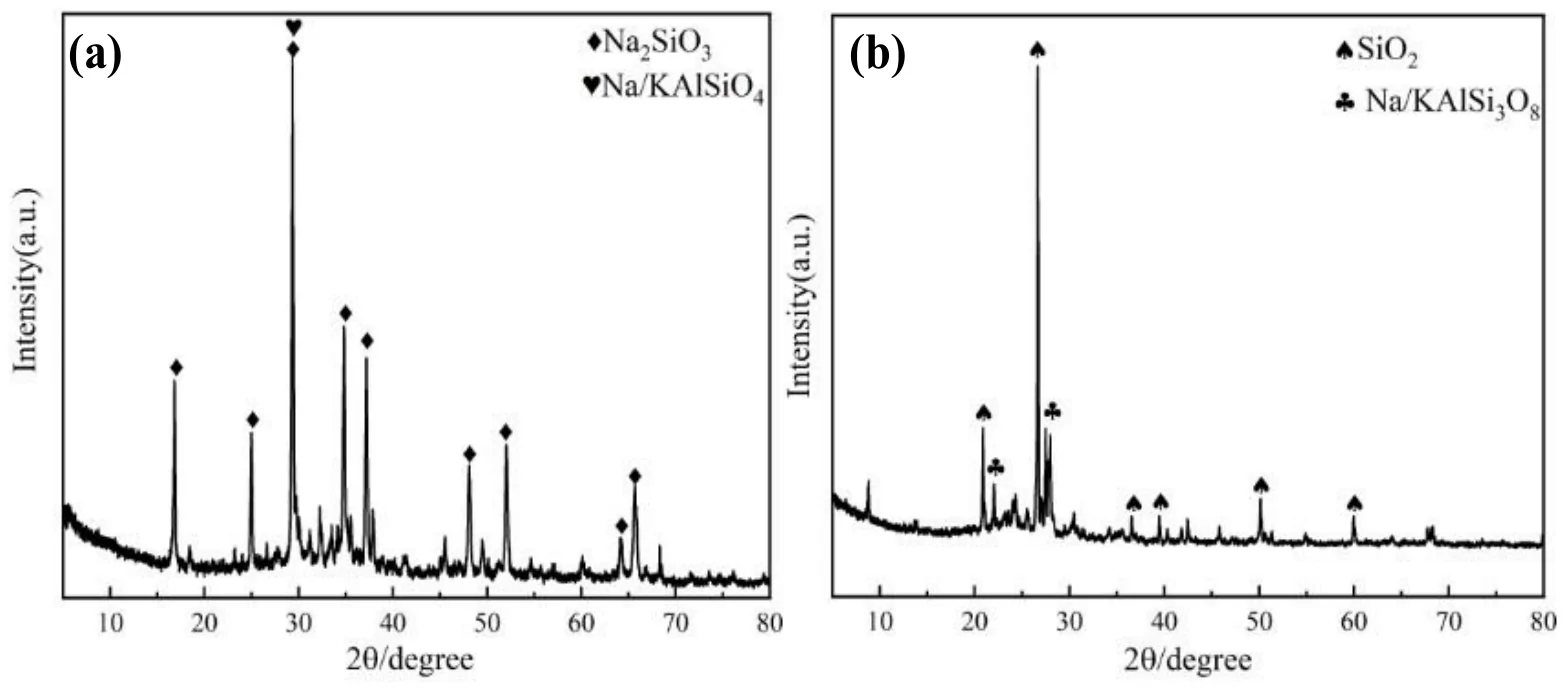
XRD patterns of products at different stages. (**a**) Activated product; (**b**) acid leaching residue.

X-ray diffraction (XRD) analyses were performed on the products and filter residues after different treatment stages. Figure 5a shows the XRD pattern after alkali fusion treatment. Compared with the XRD pattern of potassium feldspar in Figure 1, the peaks of the silico-aluminate and quartz phases have almost completely disappeared after alkali fusion treatment, while peaks for sodium silicate and potassium/sodium feldspar have appeared, indicating that silicon and aluminum elements participated in the reaction. The feldspar in the activation product underwent decomposition under acidic conditions, generating silicic acid. Concurrently, elements such as aluminum (Al), potassium (K), and sodium (Na) from the feldspar were enriched in the acid solution in the form of Al^3+^, K^+^, and Na^+^. After centrifugation and filtration, the acid leaching residue was obtained. The filtrate consists of partially unreacted SiO_2_ (as shown in Figure 5).

Based on the XRD test results, it can be concluded that during the calcination of potassium feldspar in a sodium hydroxide-based alkaline melt, the primary chemical reactions between potassium feldspar and sodium hydroxide are as shown in Equations (1)–(3). The main calcination products are KAlSiO_4_, NaAlSiO_4_, and Na_2_SiO_3_.

(1) $${\mathrm{KAlSi}}_{3}O_{8}+4\mathrm{NaOH}={\mathrm{KAlSiO}}_{4}+2{\mathrm{Na}}_{2}{\mathrm{SiO}}_{3}+2H_{2}O$$

(2) $${\mathrm{NaAlSi}}_{3}O_{8}+4\mathrm{NaOH}={\mathrm{NaAlSiO}}_{4}+2{\mathrm{Na}}_{2}{\mathrm{SiO}}_{3}+2H_{2}O$$

(3) $${\mathrm{SiO}}_{2}+2\mathrm{NaOH}={\mathrm{Na}}_{2}{\mathrm{SiO}}_{3}+2H_{2}O$$

During the acid leaching process, the reaction primarily involves feldspar reacting with hydrochloric acid, as shown in chemical Equations (4)–(6). The chemical composition of the leachate consists mainly of H_2_SiO_3_, AlCl_3_, NaCl, and trace amounts of metal ions.

(4) $${\mathrm{KAlSiO}}_{4}+4\mathrm{HCl}=\mathrm{KCl}+{\mathrm{AlCl}}_{3}+H_{2}{\mathrm{SiO}}_{3}+{\;H}_{2}O$$

(5) $${\mathrm{NaAlSiO}}_{4}+4\mathrm{HCl}=\mathrm{NaCl}+{\mathrm{AlCl}}_{3}+{\;H}_{2}{\mathrm{SiO}}_{3}+H_{2}O$$

(6) $${\mathrm{Na}}_{2}{\mathrm{SiO}}_{3}+2\mathrm{HCl}=H_{2}{\mathrm{SiO}}_{3}+2\mathrm{NaCl}$$

The full evolution of Si and Al species throughout the pretreatment process can be summarized as follows. During alkali fusion, hydroxide ions attack and break the bridging Si-O-Si and Si-O-Al bonds in the rigid three-dimensional framework of potassium feldspar, depolymerizing the crystalline mineral into soluble sodium silicate and alkali metal aluminosilicate phases. In the subsequent acid leaching step, silicate species react with H^+^ to form highly dispersed orthosilicic acid (H_4_SiO_4_) monomers, while aluminosilicate phases dissociate to release free Al^3+^ ions. This achieves molecular-level uniform mixing of Si and Al species in the leachate, which provides the prerequisite for homogeneous formation of Si-O-Al bonds in the following sol–gel stage.

### 2.2. Effect of Propylene Oxide Content on Gel Formation

In the preparation of SiO_2_-Al_2_O_3_ aerogel, a silicon- and aluminum-rich acid leachate was used as the precursor, while propylene oxide (PO) serves as the cross-linking agent. The oxygen atoms in PO are strong nucleophiles, enabling them to attack protonated species and drive the ring-opening reaction. In the reaction system, chloride ions (Cl^−^) act as even stronger nucleophiles than water and preferentially attack and open the ring of protonated epoxides (as shown in Equation (7)). Because this ring-opening with Cl^−^ is irreversible, it leads to a net reduction in the number of protons in solution [42]. PO is a weak base, and, as the reaction progresses, both PO and water are consumed, resulting in a slow and uniform increase in the solution’s ph. This gradual pH rise initiates condensation in the sol, leading to the formation of small oligomer particles. These particles aggregate into larger clusters, which then interconnect within the medium, forming an extensive network and ultimately resulting in gel formation [43,44,45]. The gradual process helps alleviate internal stresses generated during gel formation and yields a network with uniform structure. Generally, the interaction between anions and metal systems is determined by the electronegativity of the anions relative to water molecules [46]. The non-coordinating nature of Cl^−^ prevents it from participating in colloidal reactions. An appropriate amount of PO causes the interionic polymerization to transition from chain-like to ring-like polymerization, thereby reducing the stress generated by dehydration condensation between hydroxyl groups within the pores and maintaining the gel’s network structure.


(7)

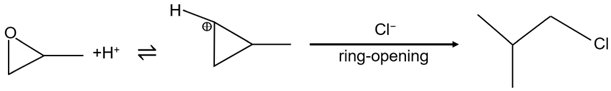



Experiments have shown that adding too much PO results in rapid gelation, producing a loose and structurally unstable gel, whereas adding too little PO prolongs the gelation process, requiring a longer waiting time. Therefore, after careful consideration and repeated testing, the optimal amount of PO was determined to be the amount that brings the pH of the acid solution to 3–4. This method was used to prepare SiO_2_-Al_2_O_3_ aerogel, and a comparative analysis of their various properties was conducted.

Different from direct alkali addition which causes local over-alkalinity and rapid uneven particle aggregation, PO consumes protons via irreversible ring-opening reaction to realize a uniform and slow rise in system pH, thus precisely regulating the polycondensation kinetics of Si-OH and Al-OH species. A moderate pH rising rate promotes homogeneous nucleation and gradual polycondensation, forming colloidal particles with consistent size and constructing a three-dimensional porous network with good connectivity and narrow pore size distribution. Excessive PO will accelerate the pH rise rate, leading to rapid particle agglomeration and an inhomogeneous macroporous structure, which is consistent with our experimental observations [47].

### 2.3. Properties of SiO_2_-Al_2_O_3_ Aerogel

Acid solutions were prepared using hydrochloric acid at various concentrations, and their pH was adjusted to 3–4 by adding PO to prepare aerogel. A systematic study was conducted to investigate the effect of hydrochloric acid concentration on the properties of SiO_2_-Al_2_O_3_ aerogel.

#### 2.3.1. Morphology of SiO_2_-Al_2_O_3_ Aerogel

**Figure 6:**
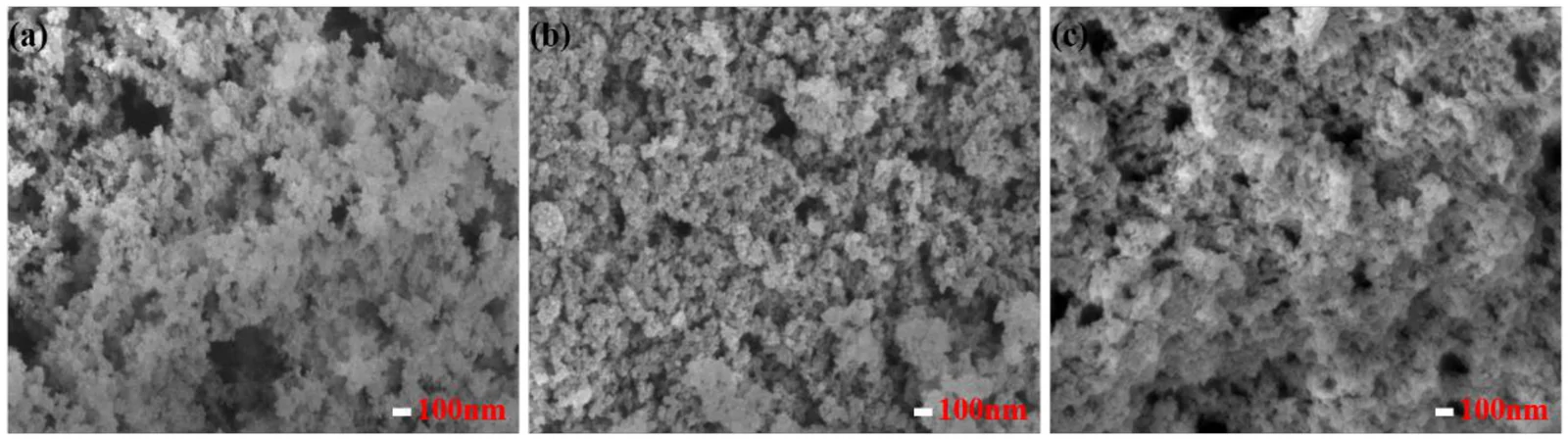
SEM images of SiO_2_-Al_2_O_3_ aerogels prepared using different acid leaching solutions (**a**) 3M, (**b**) 4M, (**c**) 5M.

Figure 6 shows scanning electron microscope (SEM) images of SiO_2_-Al_2_O_3_ aerogels prepared using different acid solutions. All SEM images exhibit typical microstructural characteristics of aerogels. At a hydrochloric acid concentration of 3M, the aerogel particles are relatively large and unevenly distributed, with a relatively loose skeletal structure. Significant particle agglomeration is observed, forming larger cluster structures. While there are many large pores, the pore connectivity is relatively poor. At the concentration of 4M, the microstructure of the aerogel was significantly improved: particle size decreased markedly, distribution became more uniform, the skeletal structure was denser and more uniform, the three-dimensional network was well-developed, particle dispersion was optimal, agglomeration was significantly reduced, pore size distribution was relatively uniform, the proportion of mesopores increased, and pore connectivity was markedly improved. Under these conditions, the aerogel network structure is ideal. Hydrochloric acid at a concentration of 4M allows the water-soluble slag to dissolve fully, with silicon and aluminum dissolving uniformly in the liquid at an appropriate rate. The precursor solution exhibits good homogeneity, enabling the formation of a uniform nanoscale network framework during the propylene oxide-induced gelation process. At 5M, the aerogel exhibits signs of over-refinement and localized structural deterioration. Particle size decreases further, but the skeletal structure becomes slightly disordered with visible irregular aggregates and regional densification or collapse. The skeletal structure tends to become dense and slightly disordered, with irregular aggregates visible in some areas, and structural collapse or densification occurring in certain regions [48].

**Figure 7:**
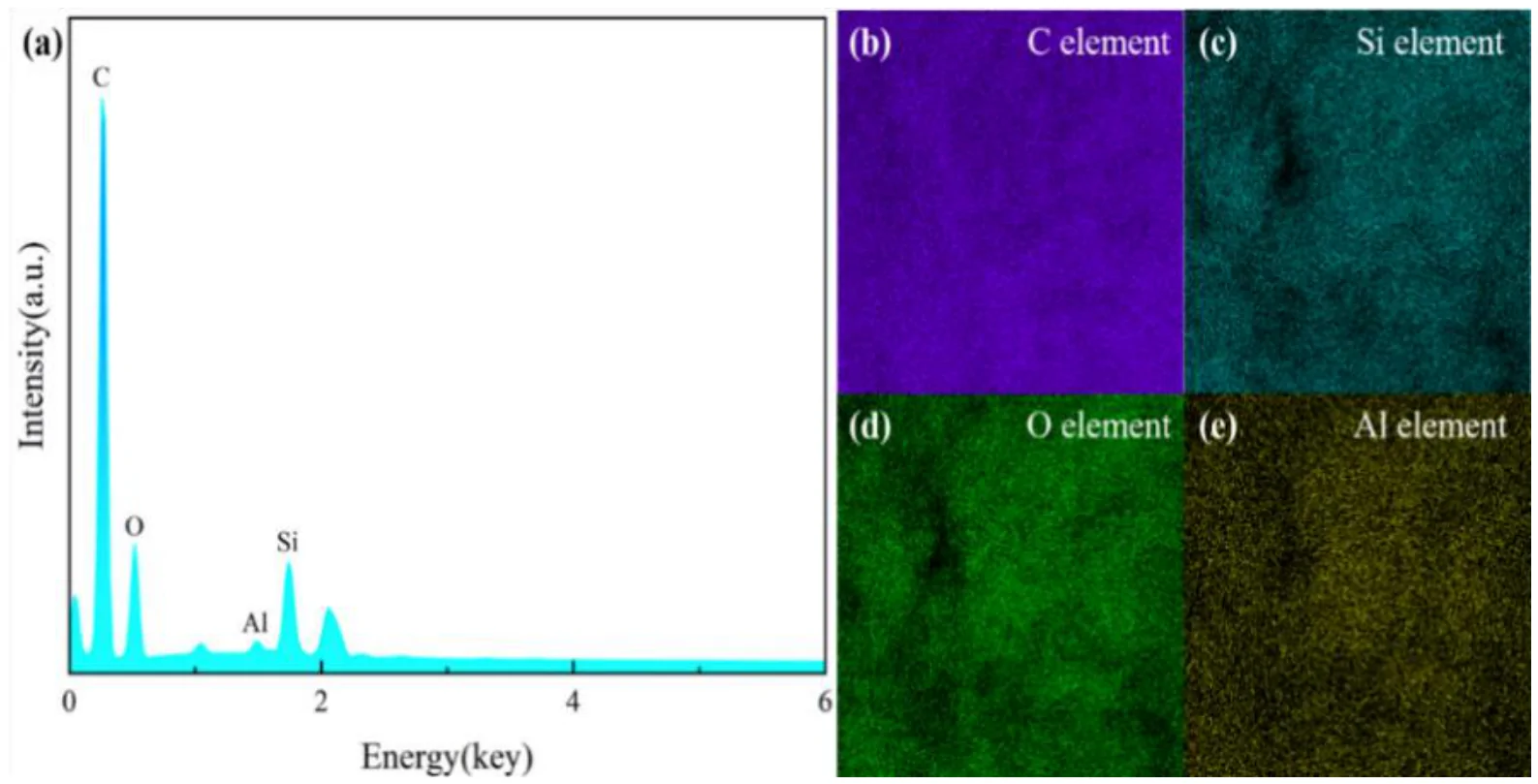
(**a**) EDS spectrum of the SiO_2_-Al_2_O_3_ aerogel EDS elemental mapping: (**b**) C, (**c**) Si, (**d**) O, and (**e**) Al.

Figure 7 shows the EDS spectrum of the SiO_2_-Al_2_O_3_ aerogel. Figure 7a reveals a characteristic peak for aluminum at approximately 1.487 keV. Combined with the spatial distribution of aluminum and silicon shown in Figure 7e, which indicates a high degree of spatial overlap and uniform dispersion-this confirms that aluminum has been successfully incorporated into the silica network structure through chemical bonding, forming a silicon–aluminum composite aerogel framework based on Si-O-Al bonds, rather than through physical doping or surface contamination.

#### 2.3.2. Microstructure of SiO_2_-Al_2_O_3_ Aerogel

**Figure 8:**
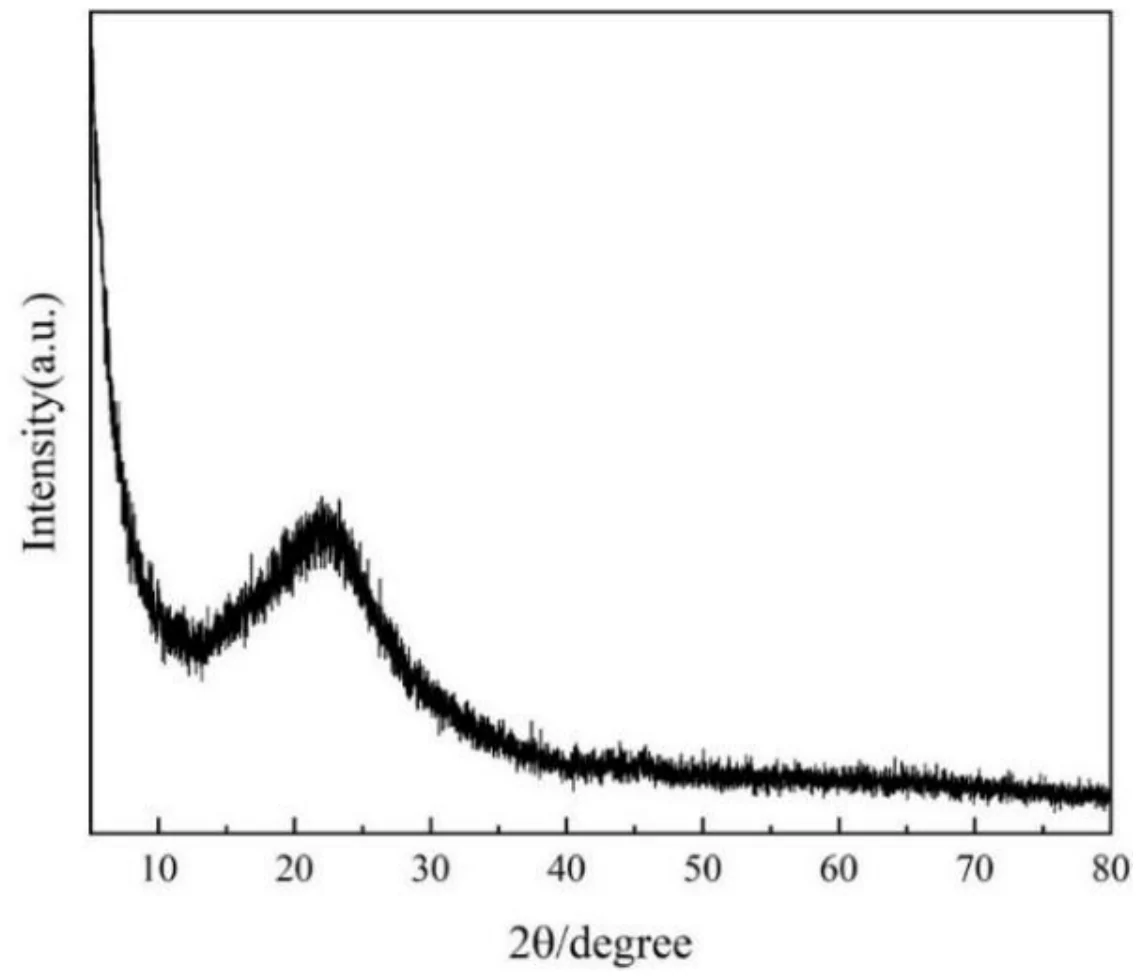
XRD pattern of SiO_2_-Al_2_O_3_ aerogel.

Figure 8 shows the X-ray diffraction pattern of the SiO_2_-Al_2_O_3_ aerogel. A diffuse diffraction peak is observed in the range of approximately 10° to 30° in the 2θ direction, which is a characteristic diffraction peak of amorphous silica, indicating that the sample consists primarily of an amorphous structure. However, no distinct characteristic diffraction peak for alumina is observed in the pattern. This is likely due to the formation of Si-O-Al bonds during the sol–gel process, whereby aluminum atoms incorporate into the silicon-oxygen network framework, preventing the formation of an independent crystalline phase of alumina and thus making its diffraction signal undetectable. Furthermore, in aerogel prepared from potassium feldspar, the Si/Al ratio is typically high. As a minor component, the scattering signal of aluminum oxide is masked by the extensive diffuse peak of amorphous silica.

**Figure 9:**
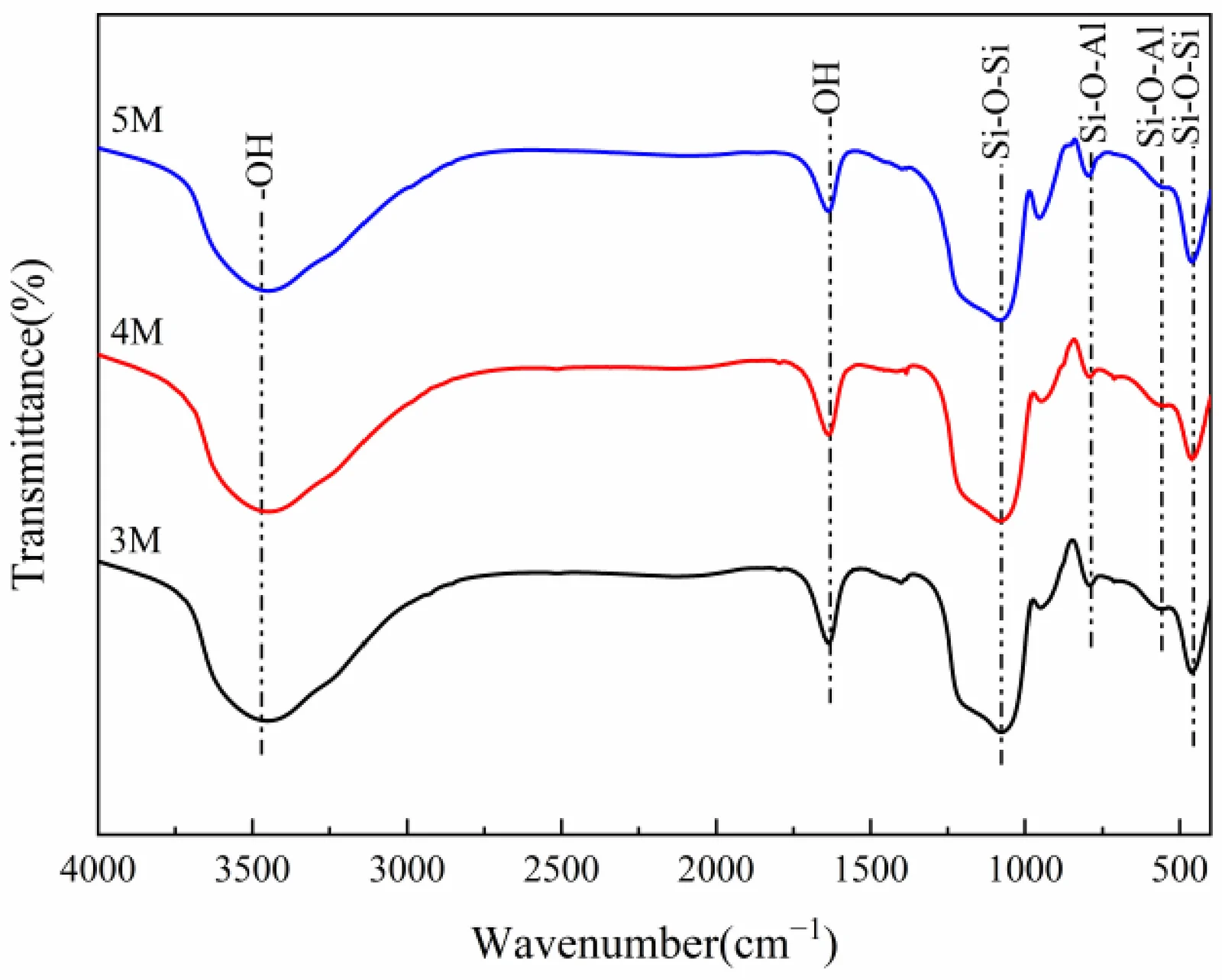
FTIR spectrum of SiO_2_-Al_2_O_3_ aerogel.

Figure 9 shows the infrared spectra of aerogels prepared using different acid solutions. The absorption peaks at 3460 cm^−1^ and 1638 cm^−1^ correspond to the deformation vibrations of the -OH group, which are primarily attributed to adsorbed water and hydroxyl groups attached to the gel surface. The absorption peaks at 1072 cm^−1^ and 462 cm^−1^ are caused by the asymmetric, symmetric, and bending vibrations of the Si-O-Si bond. The peaks at 794 cm^−1^ and 570 cm^−1^ correspond to the Si-O-Al bond, which is consistent with the conclusions reached by Shen et al. [35], indicating that the prepared aerogel is a silicon–aluminum composite aerogel, and that the absorption peaks of aerogels prepared with hydrochloric acid of different concentrations are almost identical.

**Figure 10:**
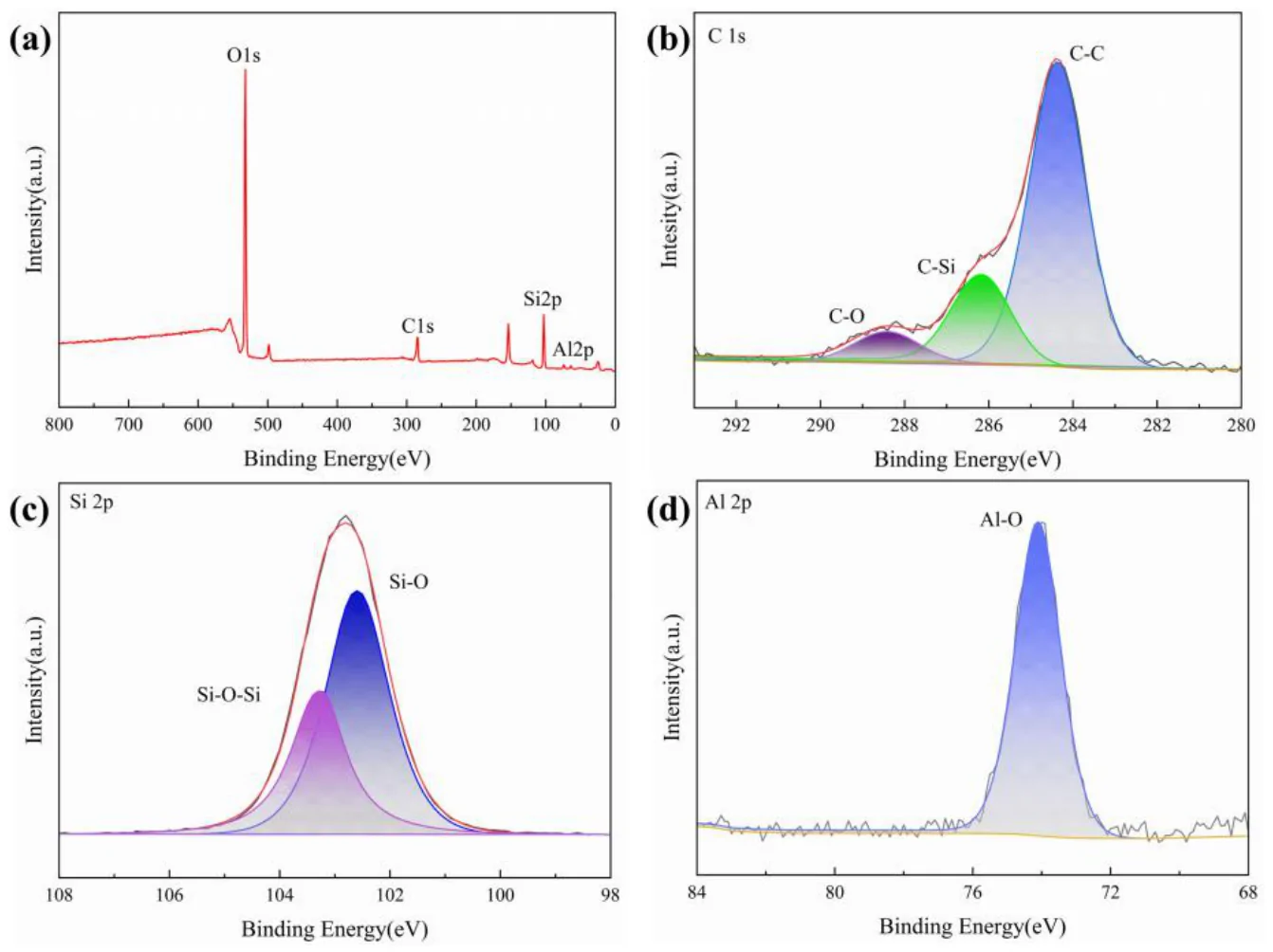
(**a**) XPS full spectrum of the SiO_2_-Al_2_O_3_ aerogel, (**b**) C1s, (**c**) Si2p and (**d**) Al2p high-resolution spectra.

Figure 10a shows the high-resolution XPS total spectrum of the aerogel, revealing peaks corresponding to C1s, Si2p, and Al2p. The C1s spectrum reveals peaks characteristic of C-O, C-Si, and C-C functional groups, with binding energies of 288.42, 286.18, and 284.8 eV, respectively. In the high-resolution Si2p spectrum, the Si-O-Si peak is located at 103.35 eV, and the Si-O peak is located at 102.6 eV. The Al2p spectrum reveals a characteristic Al-O functional group with a binding energy of 74.1 eV [49,50,51], corresponding to oxygen-coordinated aluminum species. The uniform single peak profile demonstrates that aluminum has a homogeneous chemical environment within the framework. Aluminum atoms are incorporated into the siloxane network via Al-O bonds to form Si-O-Al hybrid structures, with no detectable characteristic peaks of isolated crystalline alumina or metallic aluminum. This result directly validates the uniform integration of silicon and aluminum components, which serves as the structural basis for the remarkable enhancement in the material’s thermal stability.

From the perspective of chemical bonding, the XPS results confirm that the as-prepared aerogel features a hybrid amorphous network cross-linked by Si-O-Al bonds, where aluminum is uniformly distributed in the silica framework rather than existing as independent phases. A certain content of hydroxyl groups is retained on the aerogel surface, which is consistent with the structural characteristics of sol–gel-derived oxide aerogels. Such chemical structural features provide fundamental support for the material’s outstanding high-temperature structural stability, as well as its application potential in adsorption and catalysis.

Combined with FTIR, XPS and EDS results, the absence of independent alumina phases is mainly attributed to the formation of Si-O-Al bonds, where Al atoms are incorporated into the silica network at the atomic level rather than forming separate phases. Specifically, FTIR detected characteristic Si-O-Al absorption peaks at 794 cm^−1^ and 570 cm^−1^; the Al2p binding energy in XPS corresponds to the Al-O structure rather than independent Al_2_O_3_; EDS elemental mapping further confirmed the uniform dispersion of Al and its high spatial coincidence with Si, excluding local aggregation of amorphous alumina phases. Although the high Si/Al ratio of the sample will also weaken the diffraction signal of Al-containing components, the multi-evidence results support that Al has been embedded into the silica framework via chemical bonding.

#### 2.3.3. Specific Surface Area and Pore Size of SiO_2_-Al_2_O_3_ Aerogel

**Figure 11:**
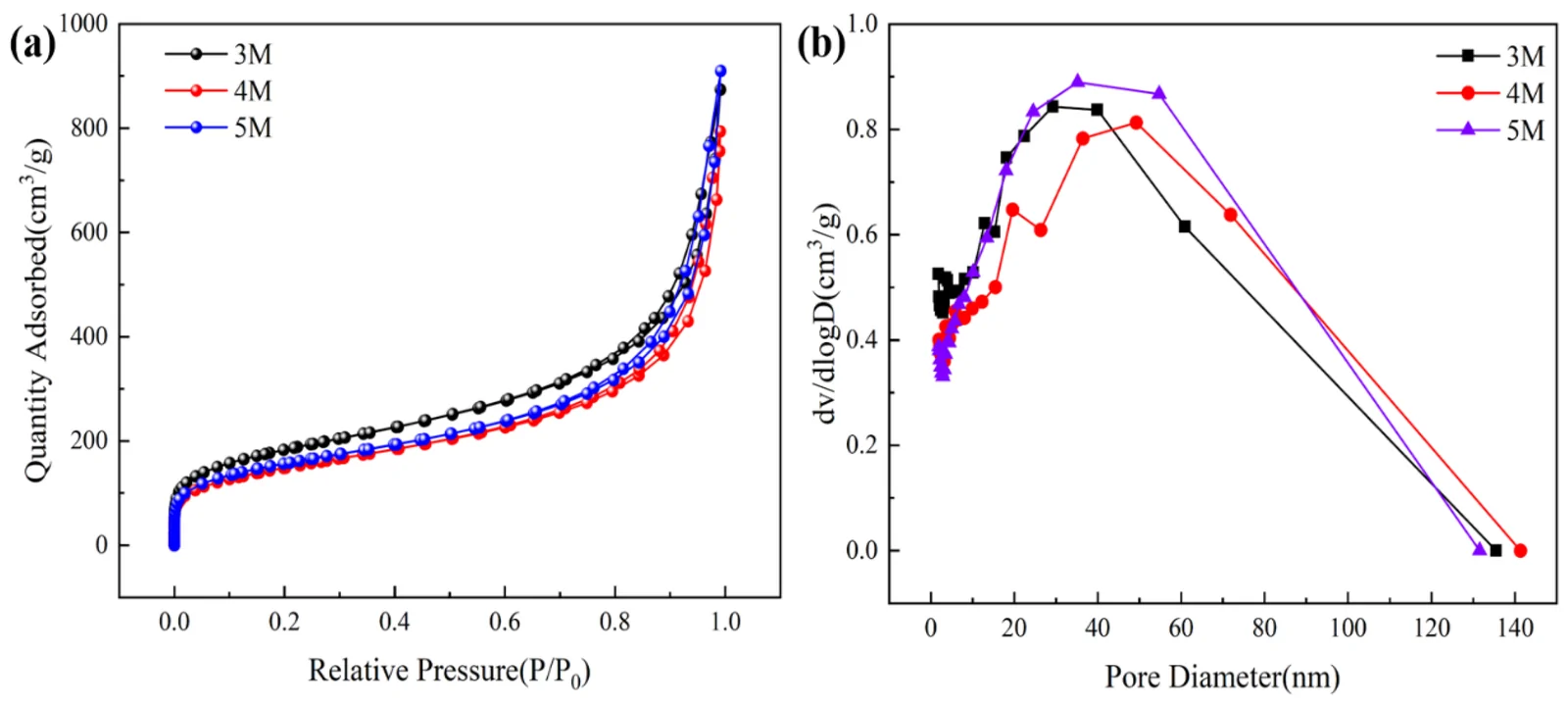
(**a**) Nitrogen adsorption–desorption curves and (**b**) pore size distributions of SiO_2_-Al_2_O_3_ aerogels prepared using different acid leaching solutions.

BET measurements were performed on SiO_2_-Al_2_O_3_ aerogels prepared using acid solutions with hydrochloric acid concentrations of 3, 4 and 5M. The results are shown in Figure 11. As shown in Figure 11a, the adsorption–desorption isotherms of the samples belong to Type IV, consistent with the characteristics of aerogel adsorption–desorption isotherms. The pore size distribution in Figure 11b indicates that the pore sizes of the prepared aerogels are predominantly distributed between 2 nm and 60 nm, and are consistent with those of the SiO_2_-Al_2_O_3_ aerogel prepared by Yu et al. [52] using AlCl_3_·6H_2_O and TEOS. Furthermore, the concentration of hydrochloric acid also has a certain influence on the pore structure of the aerogel. The 3M sample exhibited the highest pore volume distribution in the 2–10 nm range and maintained a high pore volume level in the 10–60 nm range. This broad distribution is consistent with the morphology observed via SEM, which revealed particle agglomeration and a high abundance of large pores. Although the abundant mesopores and macropores provide a high specific surface area, the relatively large pore sizes and uneven distribution result in a higher heat transfer efficiency of gas molecules within the pore channels. In contrast, the pore size distribution of the 4M sample is relatively concentrated, primarily within the 2–40 nm range, with a peak in the smaller pore size region, and the overall pore volume is significantly lower than that of the 3M sample. The pore size distribution curve of the 5M sample is similar to that of the 4M sample, but the pore volume in the micropore and smaller mesopore regions is slightly higher than that of the 4M sample, while the pore volume in the larger mesopore region is lower. This distribution characteristic indicates that more micropores and smaller mesopores were formed at higher acid concentrations, while the formation of large pores was suppressed. An increase in the proportion of micropores helps reduce gas thermal conductivity because when the pore size is smaller than the average free path of gas molecules, gas heat transfer is significantly inhibited. It should be noted that trace TiO_2_ impurities remaining in the precursor have no significant effect on the evolution of pore structure, and the change in pore structure with acid concentration is dominated by the dissolution and polymerization behavior of silicon and aluminum species.

**Figure 12:**
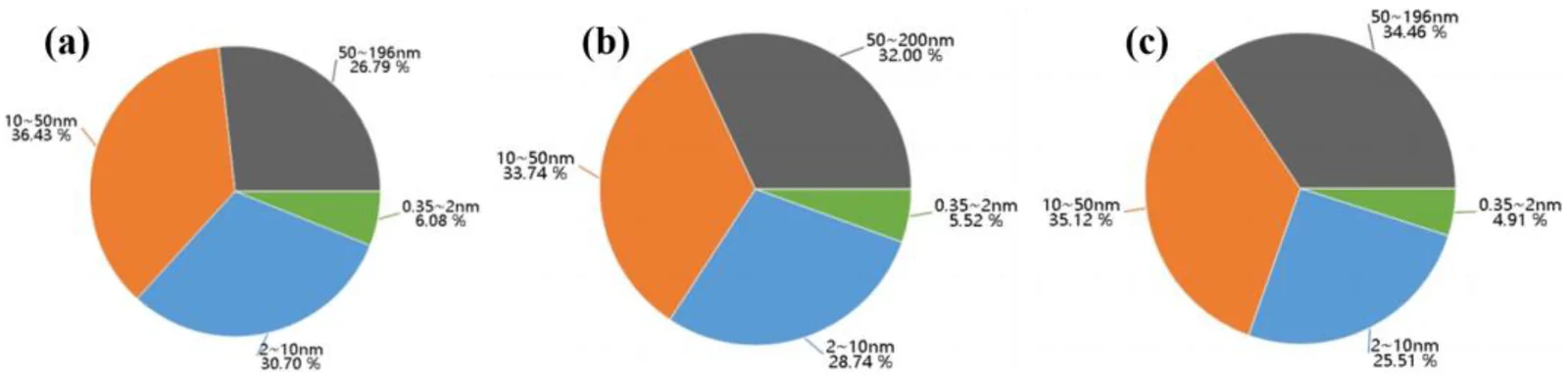
BJH pore size distribution curves of the as-prepared SiO_2_-Al_2_O_3_ aerogels prepared with different HCl concentrations: (**a**) 3M, (**b**) 4M, and (**c**) 5M.

As shown in Table 3 and Figure 12, the highest specific surface area of the 3M sample is attributed to its highest micropore proportion. Under 3M HCl, the moderate proton concentration leads to a mild polycondensation rate of Si-OH and Al-OH groups, facilitating uniform molecular-level cross-linking and generating abundant micropores that contribute significantly to the specific surface area. When HCl concentration increases to 4M, the enhanced cross-linking degree of the Si-O-Al network compresses the internal pore structure, resulting in the lowest total pore volume and reduced micropore content, thus decreasing the specific surface area. As the concentration further rises to 5M, the excessively fast polycondensation rate drives severe aggregation of colloidal particles, forming a large number of inter-particle mesopores. This leads to the maximum total pore volume and larger average pore size, while the reduced micropore content keeps its specific surface area far lower than that of the 3M sample.

#### 2.3.4. Thermal Stability of SiO_2_-Al_2_O_3_ Aerogel

**Figure 13:**
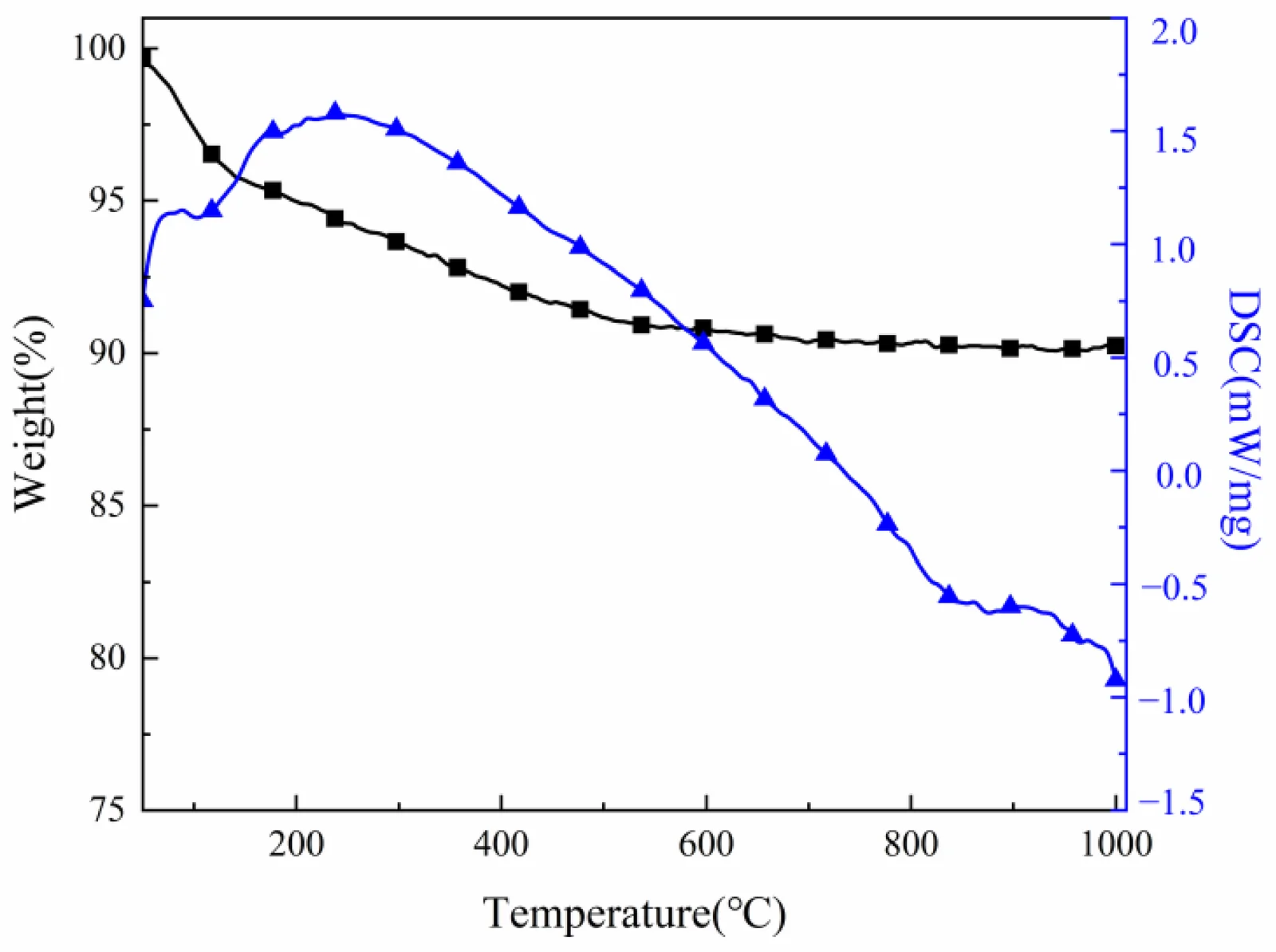
TG-DSC curves of SiO_2_-Al_2_O_3_ aerogel.

Thermogravimetric analysis was performed on the prepared SiO_2_-Al_2_O_3_ aerogel samples at temperatures ranging from room temperature to 1000 °C. The results are shown in Figure 13. As shown in the figure, within the temperature range from room temperature to 1000 °C, mass loss primarily occurred in two distinct regions. The first region of mass loss spans from room temperature to 200 °C, with a corresponding mass loss of 6.60%. The endothermic feature in this region corresponds to the volatilization of adsorbed water and residual ethanol. The second weight loss interval is from 200 °C to 1000 °C, with a corresponding mass loss of 2.95%, which corresponds to the oxidative decomposition of residual organic matter and a small amount of Al-OR groups. Consistent with this slow mass loss, a broad and weak exothermic peak appears on the DSC curve in the 200–650 °C range. No distinct endothermic or sharp exothermic peaks are observed in the high-temperature region above 650 °C. In particular, no characteristic sharp exothermic peak of mullite crystallization is detected in the 900–1000 °C range, and no stepwise mass change occurs on the TG curve simultaneously. This indicates that no significant thermal events such as mullite crystallization, alumina phase transition, or severe framework sintering occur in the aerogel below 1000 °C. The uniformly embedded Al atoms in the silica network via Si-O-Al bonds restrict the diffusion of Al atoms and the nucleation of crystalline phases, thus effectively suppressing high-temperature phase transition and structural collapse. Table 4 compares the thermal stability of different SiO_2_-Al_2_O_3_ aerogels. In addition, the trace TiO_2_ introduced from the raw material does not cause deterioration of thermal stability, and may even slightly enhance the high-temperature resistance of the framework through chemical bond doping, which also contributes to the low high-temperature mass loss of the sample.

**Figure 14:**
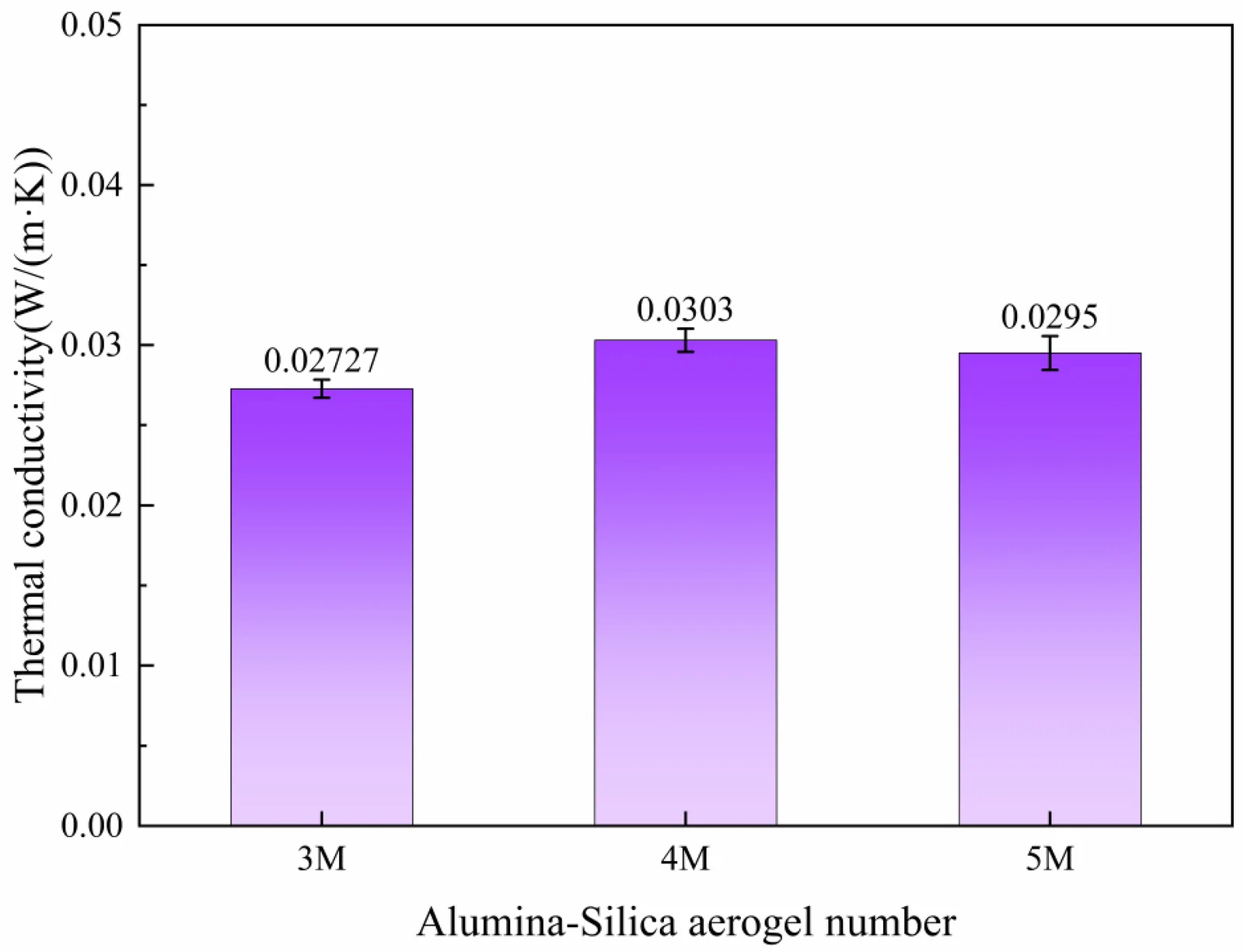
Thermal conductivity profiles of the as-prepared SiO_2_-Al_2_O_3_ aerogels.

The as-prepared SiO_2_-Al_2_O_3_ aerogels feature a high specific surface area, an ultra-low thermal conductivity of 0.027 W/(m·K) (Figure 14), and excellent structural stability below 1000 °C, demonstrating great application potential in a wide range of fields. For high-temperature thermal insulation, its stable porous structure and the inherent low thermal conductivity of amorphous oxide aerogels make it a promising candidate for thermal protection scenarios above 800 °C. As a catalyst support, the uniformly dispersed Al active sites and high specific surface area can provide abundant loading sites for catalytic active components, while its outstanding thermal stability can maintain structural integrity under high-temperature reaction conditions. In the field of environmental remediation, abundant surface hydroxyl groups and the porous structure endow the material with good adsorption capacity for heavy metal ions and organic pollutants in wastewater [61,62,63].

## 3. Conclusions

We proposed a method for preparing SiO_2_-Al_2_O_3_ composite aerogel from potassium feldspar via alkali fusion activation–acid leaching–sol–gel synthesis coupled with supercritical drying. Optimal alkali fusion conditions of 350 °C for 120 min with a mass ratio of potassium feldspar to sodium hydroxide of 1:1.2 achieved a reaction rate of 97.3%, enabling efficient extraction of silicon and aluminum. The acid leachate was gelled by adjusting the pH to 3–4 using propylene oxide as a gelation promoter. After aging, solvent exchange, and supercritical drying, SiO_2_-Al_2_O_3_ aerogel was obtained. Characterization results confirmed that aluminum was incorporated into the siloxane network through Si-O-Al bonds to form a composite framework, rather than by physical doping, with uniform elemental distribution. The resulting SiO_2_-Al_2_O_3_ aerogel exhibits a three-dimensional nanoporous network with a specific surface area of 660.841 m^2^/g, pore volume of 1.321 cm^3^/g. The mass loss is only 9.55% in the temperature range of 0–1000 °C, demonstrating significantly superior thermal stability compared to pure alumina aerogel. This study provides a viable approach for the high-value utilization of potassium feldspar and the low-cost preparation of silica–alumina aerogel. In subsequent work, we will further carry out high-precision characterization of trace impurity phases and explore their effects on the optical properties and long-term thermal stability of aerogels, to provide a more comprehensive theoretical basis for the high-value utilization of potassium feldspar.

## 4. Materials and Methods

### 4.1. Materials

The potassium feldspar was sourced from Hubei, China. Sodium hydroxide, hydrochloric acid, anhydrous ethanol, and propylene oxide were purchased from Shanghai Sinopharm Chemical Reagent Co., Ltd., Shanghai, China, all of analytical grade. Deionized water was prepared using laboratory equipment.

### 4.2. Activation of Potassium Feldspar and Preparation of SiO_2_-Al_2_O_3_ Aerogel

(1) After impurities are removed from potassium feldspar using sulfuric acid, dry the powder at 60 °C for 6 h to evaporate surface moisture, then sieve it through a 180-mesh sieve to obtain potassium feldspar powder.

(2) The potassium feldspar powder is mixed with NaOH in various mass ratios in a crucible until uniform, then calcine and activate the mixture in a muffle furnace at a heating rate of 10 °C/min for a specified duration. After calcination, samples are naturally cooled via the muffle furnace’s built-in cooling system and taken out only when the furnace temperature drops below 50 °C.

(3) The active produce is ground and sieved. It is then mixed with deionized water in beaker at the ratio of 1 g to 4 mL. After stirring to disperse the mixture, add hydrochloric acid solutions of varying concentrations at a ratio of 1 g to 8 mL. Stir at 540 r/min at room temperature to ensure the activated product reacts and dissolves completely. After stirring for 2 h, filter the mixture to separate the residue from the filtrate. Collect the filtrate and dry the residue to determine its mass.

(4) Under stirring conditions, propylene oxide (PO) is added dropwise to the filtrate. A dosage of 0.1–0.3 mL PO is added per milliliter of leachate, i.e., 0.1–0.3 mL PO per 1 mL leachate. The pH of the hydrochloric acid leachate is approximately 1 after acid leaching. Upon PO addition, the pH of the acid leachate rises slowly to 3–4. Gelation occurs after standing for a certain period, yielding silica–alumina gel. The gel is immersed in deionized water and placed in water bath maintained at 45 °C for 24 h to cure.

(5) The aged gel is immersed in anhydrous ethanol in 45 °C water bath for 24 h, replacing the ethanol every 4 h to fully displace the water molecules within the gel. Finally, supercritical CO_2_ drying is performed at 50 °C and 12 MPa with a 12 h pressure holding period to obtain the SiO_2_-Al_2_O_3_ aerogel sample.

### 4.3. Testing and Characterization

KR represents the acid-leaching efficiency of potassium feldspar after alkali fusion activation, which is used to indirectly characterize the decomposition degree of potassium feldspar. It is calculated by the formula KR = (m − m_0_)/m, where m is the initial mass of raw potassium feldspar, and m_0_ is the mass of insoluble filter residue after water dispersion and hydrochloric acid leaching. The residue mainly consists of unreacted quartz, incompletely decomposed feldspar and other acid-insoluble inert impurities. The morphology and structure of the aerogel were characterized using a field-emission scanning electron microscope equipped with a UITIM Max 65 (JEOL Ltd., Tokyo, Japan) energy dispersive spectrometer (EDS), and elemental analysis was performed using the attached EDS. An X-ray diffractometer was used to identify and characterize the phases of potassium feldspar and the activation products. A synchronous thermal analyzer was used to measure the TG, DSC, and DTG profiles of the aerogel from room temperature to 1000 °C under an air atmosphere at a heating rate of 10 °C/min. A Fourier transform infrared spectrometer was used to characterize the functional groups of the aerogel. An X-ray photoelectron spectrometer was employed to identify characteristic peaks corresponding to the types of elements and their chemical states. A fully automated specific surface area and porosity analyzer calculates the total specific surface area using the BET multi-layer adsorption model and employs microthermodynamic methods such as BJH to analyze pore volume and pore size distribution in the mesopore to micropore range, enabling quantitative characterization of the surface accessibility and pore structure of porous materials. All experimental data are averaged values from multiple repeated tests.

## Figures and Tables

**Table 1:** Major Chemical Composition of Raw Potassium Feldspar.

Elements	SiO_2_	Al_2_O_3_	K_2_O	Na_2_O	TiO_2_	Fe_2_O_3_	Others
wt (%)	72.42	15.11	4.45	4.22	0.17	1.20	2.43

**Table 2:** Major chemical composition of potassium feldspar after sulfuric acid treatment.

Elements	SiO_2_	Al_2_O_3_	K_2_O	Na_2_O	TiO_2_	Fe_2_O_3_	Others
wt (%)	75.24	14.92	4.77	4.31	0.12	0.15	2.49

**Table 3:** Specific Surface Area, Pore Volume, and Average Pore Diameter of SiO_2_-Al_2_O_3_ Aerogels Prepared by Acid Leaching with Different Concentrations of Hydrochloric Acid.

Sample	Specific Surface Area (m^2^/g)	Pore Volume (cm^3^/g)	Average Pore Diameter (nm)	Micro/Mesopore Ratios
3M	660.841	1.321	7.994	0.091
4M	535.893	1.171	8.741	0.088
5M	565.675	1.364	9.643	0.077

**Table 4:** Comparison of the Thermal Stability of Different Aerogels.

Sample	Ingredients	Temperature Range	Mass Loss	References
SiO_2_-Al_2_O_3_ aerogel	Potassium feldspar	0–1000 °C	9.55%	This work
SiO_2_-Al_2_O_3_ aerogel	Fly ash		~18%	[35]
Al_2_O_3_-SiO_2_ aerogel	AlCl_3_·6H_2_O and TEOS		14.3%	[53]
Al-modified silica aerogel	Kaolin clay		11.74%	[54]
Al_2_O_3_ aerogel	AlCl_3_·6H_2_O		~28%	[55]
SiO_2_ aerogel	fly ash and trona ore		15%	[56]
Al_2_O_3_-Pal aerogel	Palygorskite (Pal) and AlCl_3_		~28%	[57]
Al_2_O_3_-SiO_2_ aerogel	AlCl_3_·6H_2_O and TEOS		21.6%	[58]
Al_2_O_3_-SiO_2_ aerogel	AlO(OH) and TEOS		~18%	[59]
Al_2_O_3_-SiO_2_ aerogel	AlCl_3_·6H_2_O and TEOS		~30%	[60]

## Data Availability

Data will be made available on request.

## References

[1] Almeida C.M., Ghica M.E., Durães L. (2020). An overview on alumina-silica-based aerogels. Adv. Colloid Interface Sci..

[2] Peng F., Jiang Y., Feng J., Cai H., Feng J., Li L. (2021). Thermally insulating, fiber-reinforced alumina–silica aerogel composites with ultra-low shrinkage up to 1500 °C. Chem. Eng. J..

[3] Pierre A.C., Pajonk G.M. (2002). Chemistry of Aerogels and Their Applications. Chem. Rev..

[4] Kistler S.S. (1931). Coherent Expanded Aerogels and Jellies. Nature.

[5] Liu M., Huang Z., Li Z., Duan Y., Yang Y., Zhou F., Chen J., Liu Q., Wu X. (2025). Synthesis of high-performance hydrophobic silica aerogel composites with excellent flame retardant and thermal insulating properties. J. Alloys Compd..

[6] Maleki H., Durães L., Portugal A. (2014). An overview on silica aerogels synthesis and different mechanical reinforcing strategies. J. Non-Cryst. Solids.

[7] Yao J., Gao X., Wu Y., Zhao X., Li X. (2022). High-temperature resistant ambient pressure-dried aluminum doped silica aerogel from inorganic silicon and aluminum sources. Ceram. Int..

[8] Lei Z., Hengliang W., Zhang L., Yang J., Jianwei L., Jingli W. (2023). A study on preparation and properties of fly ash-based SiO_2_ aerogel material. Colloids Surf. A Physicochem. Eng. Asp..

[9] Mladenović D., Milikić J., Samancı M., Rakočević L., Santos D.M., Bayrakçeken A., Šljukić B. (2026). Tunable transition metal oxide/carbon aerogel catalysts: Insights into oxygen reduction and supercapacitor performance. J. Power Sources.

[10] Xu J., Zhang B., Lu Y., Wang L., Tao W., Teng X., Ning W., Zhang Z. (2022). Adsorption desulfurization performance of PdO/SiO_2_@graphene oxide hybrid aerogel: Influence of graphene oxide. J. Hazard. Mater..

[11] Zhu Y., Zhang T., Lv L., Tang W., Wang Y., Tang S. (2024). Design and synthesis of a macro-porous chitosan-sodium alginate aerogel adsorbent for efficient adsorption of SDBS from aqueous solutions. Sep. Purif. Technol..

[12] Zhao J., Xu Y., Kou Z., Guo J., Grunewald J. (2025). Hygrothermal performance of the building envelope insulated by the silica aerogel composites in different climates. Energy Build..

[13] He S., Wu X., Zhang X., Sun J., Tian F., Guo S., Du H., Li P., Huang Y. (2023). Preparation and properties of thermal insulation coating based on silica aerogel. Energy Build..

[14] Du F., Zhu W., Yang R., Zhang Y., Wang J., Li W., Zuo W., Zhang L., Chen L., She W. (2023). Bioinspired Super Thermal Insulating, Strong and Low Carbon Cement Aerogel for Building Envelope (Adv. Sci. 18/2023). Adv. Sci..

[15] Xiang L., He C., Pei Z., Chen T., Jiang J., Chen Z., Yu Y. (2025). Multi-scale synergistic flame-retardant composite aerogel with low thermal conductivity and mechanical robustness for passive fire protection in high-rise buildings. Constr. Build. Mater..

[16] Sun J., Yao K., Zhang R. (2024). Robust-flexible and highly-thermostable polyimide-silica aerogels with adjustable fiber skeleton structure for efficient aerospace thermal protection. Mater. Today Commun..

[17] Xi S., Wang Y., Zhang X., Cao K., Su J., Shen J., Wang X. (2023). Fire-resistant polyimide-silica aerogel composite aerogels with low shrinkage, low density and high hydrophobicity for aerospace applications. Polym. Test..

[18] Wang Y., Chen Z., Yang L., Xu K., Ai S., Zhang J., He L., Xu F., Shen X., Li M. (2026). Lightweight, strong and insulation melamine carbon foam supported SiO_2_ aerogel for aerospace thermal protection systems. Chem. Eng. J..

[19] Omranpour H., Motahari S. (2013). Effects of processing conditions on silica aerogel during aging: Role of solvent, time and temperature. J. Non-Cryst. Solids.

[20] Yang H., Wang F., Zhang H., Guo L., Hu L., Wang L., Xue D.-J., Xu X. (2020). Solution Synthesis of Layered van der Waals (vdW) Ferromagnetic CrGeTe_3_ Nanosheets from a Non-vdW Cr_2_Te_3_Template. J. Am. Chem. Soc..

[21] Liu J.-X., Shi F., Bai L.-N., Feng X., Wang X.-K., Bao L. (2013). Synthesis of TiO_2_–SiO_2_ aerogel via ambient pressure drying: Effects of sol pre-modification on the microstructure and pore characteristics. J. Sol.-Gel Sci. Technol..

[22] Babiarczuk B., Lewandowski D., Szczurek A., Kierzek K., Meffert M., Gerthsen D., Kaleta J., Krzak J. (2020). Novel approach of silica-PVA hybrid aerogel synthesis by simultaneous sol-gel process and phase separation. J. Supercrit. Fluids.

[23] Wang S., Liang B., Cui J., He J., Wu J., Mao T., Cui B., Cui J., Nie D. (2025). Ambient-pressure-dried silica aerogels modified by KH-560 for tough and hydrophobic thermal insulation: Preparation and mechanism analysis. Colloids Surf. A Physicochem. Eng. Asp..

[24] Li S., Ren H., Zhu J., Bi Y., Xu Y., Zhang L. (2017). Facile fabrication of superhydrophobic, mechanically strong multifunctional silica-based aerogels at benign temperature. J. Non-Cryst. Solids.

[25] Qian S.-Y., Zhou X.-Y., Ren L., Wang X., Yao Y., He P., Shang J., Gao C. (2025). Fabrication of high-performance co-precursor silicon-based aerogels reinforced with surface modified and grafted carbon fibers at atmospheric pressure. Ceram. Int..

[26] Zhang Y., Xiang L., Shen Q., Li X., Wu T., Zhang J., Nie C. (2021). Rapid synthesis of dual-mesoporous silica aerogel with excellent adsorption capacity and ultra-low thermal conductivity. J. Non-Cryst. Solids.

[27] Mazraeh-Shahi Z.T., Shoushtari A.M., Abdouss M., Bahramian A.R. (2013). Relationship analysis of processing parameters with micro and macro structure of silica aerogel dried at ambient pressure. J. Non-Cryst. Solids.

[28] Wenyan Li Z.S. (2022). Preparation of Hydrophobic SiO_2_ Aerogel via Ambient Pressure Drying with Water Glass. J. Nanjing Tech. Univ. (Nat. Sci. Ed.).

[29] Wang Z., Liu F., Wei W., Dong C., Li Z., Liu Z. (2023). Influence of supercritical fluid parameters on the polyimide aerogels in a high-efficiency supercritical drying process. Polymer.

[30] Guo Y., Liu A., Shen K., Chen Z., Chen S., Yang L., Yang P., Ma Z., Yang H., Feng X. (2025). Lightweight multicomponent silica fibrous aerogels prepared by freeze-drying for high-temperature thermal insulation. J. Alloys Compd..

[31] Li T., Du A., Zhang T., Ding W., Liu M., Shen J., Zhang Z., Zhou B. (2018). Efficient preparation of crack-free, low-density and transparent polymethylsilsesquioxane aerogels *via* ambient pressure drying and surface modification. RSC Adv..

[32] Wei J., Zhu P., Sun H. (2022). Ambient-Dried Silica Aerogel Powders Derived from Coal Gangue by Using One-Pot Method. Materials.

[33] Hu W., Li M., Chen W., Zhang N., Li B., Wang M., Zhao Z. (2016). Preparation of hydrophobic silica aerogel with kaolin dried at ambient pressure. Colloids Surf. A Physicochem. Eng. Asp..

[34] Bo Liu M.L. (2017). Preparation of SiO_2_-Al2O3 Composite Aerogel with High Specific Surface Area by Sol-Gel Method from Coal Gangue. CIESC J..

[35] Shen M., Jiang X., Zhang M., Guo M. (2019). Synthesis of SiO_2_–Al_2_O_3_ composite aerogel from fly ash: A low-cost and facile approach. J. Sol.-Gel Sci. Technol..

[36] Li J.-Y., Tian B.-H., Li X.-X., Wang Z., Cui L.-P., Liang D.-D., Wang S.-L., Liu Y.-H., Ou H.-A., Liang H.-X. (2023). Energy effective utilization of circulating fluidized bed fly ash to prepare silicon-aluminum composite aerogel and gypsum. Waste Manag..

[37] Li T., He G., Jiang Z., Duan Y., Liu M. (2025). Research advances and new insights into multi-source solid waste-derived ceramic foams. Sustain. Mater. Technol..

[38] Sun L., Chen X., Li C., Wu Z., Yang Z., Liang T. (2025). Mix proportion design and reaction mechanism of alkali-activated slag mortar with marine materials. Constr. Build. Mater..

[39] Incorporation of Disposed Face Mask to Cement Mortar Material: An Insight into the Dynamic Mechanical Properties. https://www.mdpi.com/2075-5309/14/4/1063.

[40] Chen B., Wang S., Li W., Zheng H., Lu Y., Qi Z., Wang Q., Zhang S., Yao J., Li Y. (2025). Investigation on the atomization mechanism of alloy powder by dual-gas nozzle and the powder defects formation. Powder Technol..

[41] Deshun Kong H.W. (2013). Experimental Study on Preparation of Sodium Silicate by Purification of Gangue and Al-kali Melting Activation. Inorg. Chem. Ind..

[42] Li X., Jiao Y., Ji H., Sun X. (2013). The Effect of Propylene Oxide on Microstructure of Zirconia Monolithic Aerogel. Integr. Ferroelectr..

[43] Zhu J., Wan C., Xu H., Liu Y., Zhuang J., Sun J., Gao X., McNally T., Huang Y., Wu D. (2021). An anchoring array assembly method for enhancing the electrical conductivity of composites of polypropylene and hybrid fillers. Compos. Sci. Technol..

[44] Yang B., Guo C., Chen S., Ma J., Wang J., Liang X., Zheng L., Liu H. (2006). Effect of Acid on the Aggregation of Poly(ethylene oxide)−Poly(propylene oxide)−Poly(ethylene oxide) Block Copolymers. J. Phys. Chem. B.

[45] Ding W., Hu Z., Zhong Y., Wu Q., Sun J., Zhu X., Cui S., Shen X. (2025). Flexible, transparent, flame-retardant cellulose/silica–aluminum composite aerogel films for thermal management. J. Non-Cryst. Solids.

[46] He F., Sui C., He X., Li M. (2015). Facile synthesis of strong alumina–cellulose aerogels by a freeze-drying method. Mater. Lett..

[47] Lu J.Y., Bu Z.Q., Lei Y.Q., Wang D., He B., Wang J., Huang W.T. (2024). Facile microwave-assisted synthesis of Sb_2_O_3_-CuO nanocomposites for catalytic degradation of p-nitrophenol. J. Mol. Liq..

[48] Xie K., Ma P., Fang Y., Yang H., Wan S., Wu Z., Shi J., Prashanth K.G., Gargarella P., Zhang L. (2025). Additive manufacturing of cryogenic chemically complex alloys with sponge bone-like reticular nanoscale superstructure. Compos. Part B Eng..

[49] Huang C., Zhao Z.-Y., Deng C., Lu P., Zhao P.-P., He S., Chen S.-W., Lin W. (2021). Facile synthesis of phytic acid and aluminum hydroxide chelate-mediated hybrid complex toward fire safety of ethylene-vinyl acetate copolymer. Polym. Degrad. Stab..

[50] Wang K., Gao W. (2025). DOPO-modified HNTs reinforced silica aerogel composites: Enhanced flame retardancy, thermal insulation, and environmental stability for silicone resins. Prog. Org. Coat..

[51] Han M., Zhai Q., Wang K., Li B., Hao R., He T., Tang Y., Ji W., Ma C., Miao Y. (2025). Bio-based phytic acid modified silica–alumina aerogels with lightweight, low-smoke, and fire-safe insulation performance. Constr. Build. Mater..

[52] Yu H., Tong Z., Yue S., Li X., Su D., Ji H. (2021). Effect of SiO_2_ deposition on thermal stability of Al_2_O_3_-SiO_2_ aerogel. J. Eur. Ceram. Soc..

[53] Chen H., Sui X., Zhou C., Wang C., Liu F. (2016). Preparation and characterization of monolithic Al_2_O_3_–SiO_2_ aerogel. J. Ceram. Soc. Jpn..

[54] Ling X., Li B., Li M., Hu W., Chen W. (2018). Thermal stability of Al-modified silica aerogels through epoxide-assisted sol–gel route followed by ambient pressure drying. J. Sol.-Gel Sci. Technol..

[55] Zhang X., Zhang R., Jin S., Hu Z., Liu Y., Jin M. (2018). Synthesis of alumina aerogels from AlCl_3_·6H_2_O with an aid of acetoacetic-grafted polyvinyl alcohol. J. Sol.-Gel Sci. Technol..

[56] Wu X., Fan M., Mclaughlin J.F., Shen X., Tan G. (2018). A novel low-cost method of silica aerogel fabrication using fly ash and trona ore with ambient pressure drying technique. Powder Technol..

[57] Zhou X., Jin H., Shen Q., Huang W., Li J., Liang Y., Mao P., Yang Y., Yun S., Chen J. (2022). Rapid preparation of Palygorskite/Al2O3 composite aerogels with superhydrophobicity, high-temperature thermal insulation and improved mechanical properties. Ceram. Int..

[58] Wu X., Shao G., Cui S., Wang L., Shen X. (2016). Synthesis of a novel Al_2_O_3_–SiO_2_ composite aerogel with high specific surface area at elevated temperatures using inexpensive inorganic salt of aluminum. Ceram. Int..

[59] Ren Y., Zhang B., Ye J., Zhong Z., Zhang J., Ye F. (2024). Preparation of monolithic Al_2_O_3_–SiO_2_ aerogels with high-temperature resistance using boehmite nanorods. Ceram. Int..

[60] Yang Z., Zhu D., Li H. (2020). A chitosan-assisted co-assembly synthetic route to low-shrinkage Al_2_O_3_–SiO_2_ aerogel via ambient pressure drying. Microporous Mesoporous Mater..

[61] Liu X.-G., Mao Q.-S., Jiang Y., Li Y., Sun J.-L., Huang F.-X. (2021). Preparation of Al_2_O_3_-SiO_2_ composite aerogels and their Cu2+ absorption properties. Int. J. Miner. Met. Mater..

[62] Huang L., Jiao X., Lin J., Hao J., Tan L., Zeng D., Wang S., Lu Y., Shen X., Zhang Z. (2026). EGDE-induced crosslinking of PEI@D500 adsorbents for direct air capture: Enhanced hydrothermal and oxidative stability. Chem. Eng. J..

[63] Accelerating Syngas-to-Aromatic Conversion via Spontaneously Monodispersed Fe in ZnCr_2_O_4_ Spinel|Nature Communications. https://www.nature.com/articles/s41467-022-33217-9.

